# Emerging Comorbidities in Adult Asthma: Risks, Clinical Associations, and Mechanisms

**DOI:** 10.1155/2016/3690628

**Published:** 2016-04-26

**Authors:** Hannu Kankaanranta, Paula Kauppi, Leena E. Tuomisto, Pinja Ilmarinen

**Affiliations:** ^1^Department of Respiratory Medicine, Seinäjoki Central Hospital, 60220 Seinäjoki, Finland; ^2^Department of Respiratory Medicine, University of Tampere, 33521 Tampere, Finland; ^3^Department of Respiratory Medicine and Allergology, Skin and Allergy Hospital, Helsinki University Hospital and Helsinki University, 00029 Helsinki, Finland

## Abstract

Asthma is a heterogeneous disease with many phenotypes, and age at disease onset is an important factor in separating the phenotypes. Most studies with asthma have been performed in patients being otherwise healthy. However, in real life, comorbid diseases are very common in adult patients. We review here the emerging comorbid conditions to asthma such as obesity, metabolic syndrome, diabetes mellitus type 2 (DM2), and cardiac and psychiatric diseases. Their role as risk factors for incident asthma and whether they affect clinical asthma are evaluated. Obesity, independently or as a part of metabolic syndrome, DM2, and depression are risk factors for incident asthma. In contrast, the effects of comorbidities on clinical asthma are less well-known and mostly studies are lacking. Cross-sectional studies in obese asthmatics suggest that they may have less well controlled asthma and worse lung function. However, no long-term clinical follow-up studies with these comorbidities and asthma were identified. These emerging comorbidities often occur in the same multimorbid adult patient and may have in common metabolic pathways and inflammatory or other alterations such as early life exposures, systemic inflammation, inflammasome, adipokines, hyperglycemia, hyperinsulinemia, lung mechanics, mitochondrial dysfunction, disturbed nitric oxide metabolism, and leukotrienes.

## 1. Introduction

Asthma is a common chronic disorder affecting more than 300 million people all over the world with prevalence among adults varying between 0.2 and 21% [1]. Recently, the cluster studies have revealed that asthma is not a single disease but a heterogeneous syndrome manifesting with distinct phenotypes. Age at asthma onset has emerged as a critical factor in distinguishing between phenotypes [2–4]. Patients with early-onset (or childhood-onset) asthma are typically atopic with Th2-predominant inflammation, good responsiveness to glucocorticoids, and good prognosis [5]. In contrast, patients with adult- or late-onset asthma are often nonatopic, are females, have less favourable prognosis, and are more likely to develop persistent airflow limitation [2–4, 6, 7]. Most of asthma is thought to start during childhood. However, this has recently been challenged by showing that, in United States, adult-onset asthma is the dominant phenotype in women from 40 years of age [8]. Adult-onset asthma and adult-onset phenotypes have been associated with factors such as female sex, obesity, occupational exposure, rhinitis, respiratory infections, smoking, stressful life events, and low level of lung function [2–4]. As recently reviewed in this journal [4] the mechanisms of adult-onset asthma may include several metabolic and inflammatory components that are common to the other diseases such as obesity, metabolic syndrome (MBO), diabetes mellitus type 2 (DM2), cardiovascular diseases (CVD), and psychiatric diseases.

Most clinical trials with asthma have been performed in patients being otherwise healthy. However, in real life, comorbid diseases are very common, especially in adult and/or aging patients. For example, in the US 35% of adult people are obese [9]. In addition, it is rather a rule than exception that patients not only often present with one comorbid disease but also have several comorbidities, that is, suffering from multimorbidity [10–12].

In early-onset childhood asthma, food allergies, atopic eczema, and allergic rhinitis are common and well-characterized comorbidities [13–16]. Some other comorbid conditions such as gastroesophageal reflux have gained a lot of attention during the years [17–19]. The role of these comorbidities has been thoroughly reviewed elsewhere [13–20]. Chronic obstructive pulmonary disease (COPD) is usually affecting age groups of 40 years and older. Asthma and COPD share many clinical features both being chronic obstructive airway diseases but having variable degrees of reversibility of airway obstruction [21]. The coexistence of asthma and COPD or the recently characterised asthma-COPD overlap syndrome (ACOS) has been reviewed and characterized in treatment guidelines of both asthma and COPD, for example, in Global Initiative for Asthma (GINA) and Global Initiative for Chronic Obstructive Lund Disease (GOLD) reports and in some national guidelines [22–26]. Thus, COPD and ACOS will not be covered by the present review.

**Figure 1:**
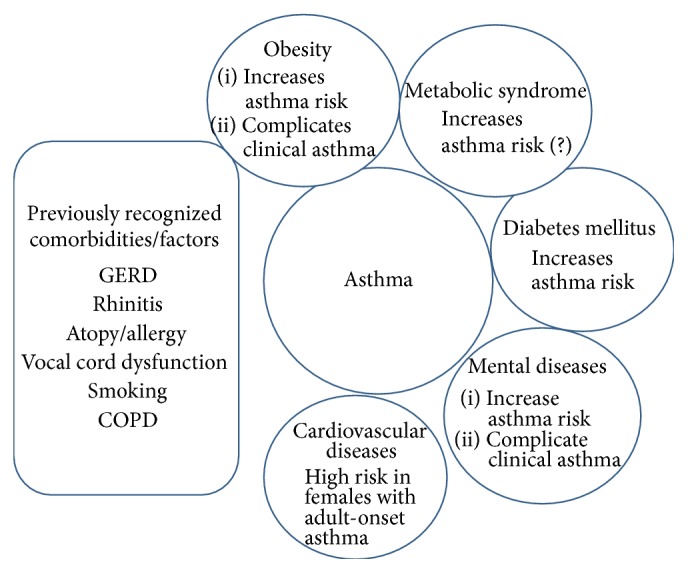
Emerging comorbidities in asthma.

In this review, we aim to give the reader an overview of the emerging comorbid conditions to asthma such as obesity, MBO, DM2, CVD, and psychiatric diseases (Figure 1). We focus on asthma in adults and especially on adult-onset asthma if evidence is available. We have five separate aims in this review if such data is available: (A) to characterize the epidemiological evidence how these comorbidities associate with asthma, (B) to describe the role of the comorbidity as a risk factor for incident asthma, (C) the possible role of asthma as a risk factor for the incident comorbidity, (D) the effect of the comorbidity on the clinical outcome of asthma, and (E) to describe the possible common mechanisms that may link asthma and the abovementioned comorbid conditions.

## 2. Epidemiologic Studies on Comorbidities in Asthma

A Norwegian study on 1 239 533 subjects of 8–29 years old comprised altogether 37 060 asthma patients when asthma was defined as using filled prescriptions on asthma drugs [27]. This study showed higher-than-expected occurrence in all of the diseases studied (attention-deficit hyperactivity disorder (ADHD), epilepsy, migraine, mental illness, cardiovascular, autoimmune, gastroesophageal reflux disease (GERD), allergy, use of antibacterials, and use of antivirals), except diabetes, in patients with asthma. Fifty-nine percent of the asthmatics had one or more of these chronic diseases when the corresponding figure for general population was 18%. In 20–29-year-olds the greatest odds ratios for other diseases in asthmatics were for allergy (OR 4.8 for men, 4.1 for women), GERD (OR 3.2 in men, 3.5 in women), and ADHD (OR 2.3 in men, 2.5 in women). The study population was 8–29 years old but still showed high prevalence of comorbidity.

In a Canadian population-based study (Ontario, Canada; population, 12 million) the burden of asthma-associated comorbidity as measured in rates of hospitalisations, emergency department visits, and ambulatory care claims was evaluated [28]. Asthma comorbidity-associated ambulatory claims, emergency department visits, and hospitalisations were substantial and, in fact, their numbers were much greater than those due to asthma itself [28]. Interestingly, the health burden due to asthma comorbidity appeared substantial regardless of age, gender, socioeconomic status, or living in a rural or urban area [28]. Approximately half of the asthma comorbidity claims were for other respiratory conditions such as upper respiratory tract infection, pneumonia, or allergic rhinitis [28]. In a further study by Gershon and coworkers [29] using the same Ontario data, comorbidity was evaluated as indicated by use of health service in 14 disease categories. In patients with asthma, comorbidity was 46% (0–4 years old), 40% (5–17 years old), 47% (18–44 years old), 49% (45–64-years old), and 22% (65 years old or more) higher than in those without asthma [29]. In all age groups most of the 14 disease categories were more common in patients with asthma. The highest comorbidity (50% or more) was found for respiratory disease other than asthma, psychiatric disorders, metabolic and immunity disorders, and perinatal disorders (0–4 years old). Risk of respiratory disease (other than asthma) was approximately double, and risk of psychiatric disorders and infectious diseases was approximately 33% to 51% higher in individuals with asthma as compared to those without asthma. In the oldest age group, physician claims were approximately twice more common for acute bronchitis and 60% higher for pneumonia in those with asthma as compared to nonasthmatics [29].

The results from Great Britain among incident cases of asthma from primary care were similar showing that patients with asthma (*n* = 7931) have more consultations in all major organs systems than their age- and gender-matched nonasthma controls [30]. The mean age of the patients with asthma in all of the abovementioned studies ranged between 30 and 32 years [28–30] suggesting that a considerable proportion of the patients represented asthma beginning during childhood. Analysis of aged (>65) patients with asthma suggests that the incidence of comorbidities increases and more closely resembles that of COPD [30]. Thus, it is likely that some comorbidity, such as allergy, may be linked to childhood-onset asthma via common genetic and environmental mechanisms. However, other comorbidities (obesity, diabetes, depression, and anxiety disorders) may be related to asthma symptoms (e.g., sleep disturbance contributing to psychiatric disorders and obesity) or its therapy, for example, glucocorticoids inducing systemic adverse effects [31], diabetes [32], or increased risk for pneumonia [33].

A German telephone interview study of 2,242 current asthmatics (1,429 women) of a total of 43,189 study individuals (18 years or older) evaluated the prevalence of eight different health conditions (diabetes mellitus, hypertension, coronary heart disease, chronic heart failure, stroke, cancer, osteoarthritis, and depression) in patients with asthma as compared with those not having current asthma [34]. All of these eight health conditions were more prevalent in patients with asthma. Almost every fifth of the patients with asthma (17.8%) had at least 3 chronic conditions whereas 8.0% of controls with no current asthma had at least 3 chronic conditions. Interestingly, multimorbidity (i.e., three or more chronic conditions) was associated with increased risk for unscheduled inpatient care (OR 3.4) and for unscheduled outpatient care (OR 2.3) [34].

An analysis of Finnish public sector employees [35] reported that asthma increased the risk of all-cause long-term (sickness absence or disability pension >90 days) work disability (HR 1.8) as compared to controls (no asthma). However, asthma and one chronic comorbid condition increased the risk for long-term all-cause work disability with HR 2.2 and asthma together with two or more chronic conditions increased the risk with HR 4.5 [35]. According to the study results asthma is a minor risk for long-term work disability but asthma with multiple comorbidities is a major risk.

Aging increases morbidity and also multimorbidity in patients with asthma [12]. By no means, multimorbidity is not a unique feature of asthma but rather represents clustering of diseases to certain persons. In fact, out of the 40 conditions studied in large analysis in Scotland [10], there was no condition in which most people had that condition only [12]. Multimorbidity is generally associated with increased risk for mental health disorders [10, 11]. Further, socioeconomic deprivation is associated with multimorbidity suggesting clustering of social, economic, and health problems in certain individuals or in certain areas. Taken together, comorbidity is common in asthma; allergy and obesity are common in younger age groups, whereas obesity, diabetes, GERD, and psychiatric and cardiovascular diseases are found in older age groups. Respiratory infections are found in all age groups.

## 3. Obesity

### 3.1. Is Obesity Associated with Asthma? Does Obesity Increase the Risk of Asthma?

The studies evaluating the association between obesity and asthma use generally body mass index (BMI) as a tool. A BMI ≥ 25 is considered overweight, while people with BMI ≥ 30 are classified as obese. BMI is particularly useful as it is standardized, calculated in the same way for both sexes and for adults of all ages [36].

Obesity is a risk factor for asthma, especially among women [37, 38]. A meta-analysis has been performed on the risk [17]. The risk for asthma was 1.20 for overweight and 1.43 for obese men. The corresponding risk estimates for women were 1.25 for overweight and 1.78 for obesity [17]. Also recent large US and Norwegian epidemiological studies report odds ratios ranging from 1.29 [39] to 1.84 [40] for obese males and 1.55 [39] to 1.96 [40] for obese females to have or to develop asthma as compared with their normal weight counterparts. What does this mean in real life? A 25-year follow-up in the Coronary Artery Risk Development in Young Adults (CARDIA) cohort gives us an approximation. Approximately 1 out of 4 obese women followed up from the age of 25 developed asthma during the 25-year follow-up period, whereas approximately 1 out of 7 normal weight women developed asthma [41].

Does increasing obesity increase the risk of asthma? A recent large US epidemiological survey reported an association with the self-reported doctor-diagnosed asthma and the class of obesity. In class I (BMI 30–34.9) obesity OR for asthma was 1.27, in class II (BMI 35–39.9) obesity OR was 1.55, and in class III (BMI ≥ 40) OR was 1.85 indicating a positive association between the degree of obesity and asthma [39]. Similarly, a large Norwegian cohort [40] reported an increased odds ratio for asthma with increasing BMI (per 5 kg/m^2^) in females (OR 1.32) and in males (OR 1.38). In the US based survey [39] a sex difference was reported by showing that class III obesity is significantly more strongly associated with asthma in women (OR 2.11) than in men (OR 1.40). Similarly, a much less pronounced risk for the development of asthma was seen between normal weight and obese men in the CARDIA study [41]. However, the effect of obesity on the development of asthma may depend on the asthma phenotype. In the long-term follow-up of children hospitalised because of bronchiolitis and many subsequently developing asthma, obesity did not affect the development of asthma in this small cohort [42].

Although cross-sectional studies or follow-up of certain cohorts in adults has demonstrated an increased prevalence of asthma in obese individuals, many of these studies rely on self-reported weight and height. Another possible limitation of these studies is that most rely on a self-reported diagnosis of asthma or symptom-based assessment of asthma rather than on physician records, physiologic evaluation, or response to inhaled therapy. This raises the possibility that the respiratory symptoms are related to the physiologic impairment from obesity rather than to asthma [43]. This is especially important with regard to obesity as obesity increases the possibility to misdiagnosis of asthma [44, 45]. For example, obese subjects who make urgent visits for respiratory symptoms are more likely to receive a misdiagnosis of asthma [44].

Taken together, it appears that obesity is a risk factor for asthma. In contrast, the role of asthma as a risk factor for obesity is much less well-known.

### 3.2. Obesity and Clinical Asthma

To evaluate the clinical association between asthma and obesity we evaluated studies that directly compared asthma-related endpoints in a cross-sectional or longitudinal study between patients (*n* ≥ 50) being normal weight or obese and reporting at least one objective measure of lung function. We identified 7 such studies [44, 46–51] together having 2597 patients with asthma (Table 1(a)). Even though most of these studies suffer from a small number of patients and exclude patients with other comorbidities, they give us an idea what the effect of obesity on asthma may be. All studies were cross-sectional and mostly included all severities of asthma. Most studies excluded smokers and patients with other comorbid states (Table 1(a)). Patients were adults (mean age between 32 and 57), but the age at the onset of the disease was not reported. However, in five studies one can assume that the population contained both patients with childhood- and adult-onset asthma (Table 1(a)). The large analysis of the Severe Asthma Research Program (SARP) [47] evaluated the effect of obesity on asthma parameters separately in early-onset (<12 years) and late-onset (≥12 years) asthma. The results are not consistent across the studies (Table 1(b)). However, the results of these studies suggest that obese patients with asthma are older (Table 1(b)). They possibly present with higher age at onset and lower control of asthma, use more steroid courses, experience more hospital admissions and emergence department visits, and have lower lung function although these differences were not reported in all of these studies. Three studies [47–49] primarily recruited patients with severe asthma. The results of these studies are more consistent with regard to oral steroid use during previous year, lung function, and blood eosinophils (Table 1(b)). In support of obese patients presenting with more severe asthma, the association of body mass index and asthma severity was evaluated in the National Asthma Survey in the US [52]. In this large (*n* = 3095) telephone survey obese asthmatics were reported to present with symptoms all the time over the past 30 days (OR 1.7), to have greater than 2 missed work days in the past 12 months (OR 1.4), to use inhaled corticosteroid (OR 1.3), to have GINA medication step 2+ asthma (OR 1.4), to present with classes II–IV persistent asthma (OR 1.4), and to present with severe persistent (class IV) asthma (OR 1.4) [52]. In this cohort, 24% (i.e., almost every fourth) of normal weight patients were classified to have moderate to severe asthma, whereas the corresponding figure among obese patients was 35% [52].

These studies (Tables 1(a) and 1(b)) suggest that there are no differences in inflammatory markers such as blood eosinophils (four studies, *n* = 1905), FENO (three studies), high-resolution computerized tomography scan (one study), and sputum inflammatory cell profiles (one study). In addition, these studies suggest that other comorbidities such as GERD, diabetes mellitus, hypertension, and obstructive sleep apnoea syndrome may be more common in obese asthma patients, although comorbidities were mostly not analyzed (Table 1(b)). Interestingly, two studies [47, 51] reported a reduced probability or tendency of being allergic when being obese and having asthma. This is in agreement with the results obtained from cluster studies that suggest that the recently identified group of patients with adult-onset obese noneosinophilic asthma phenotype are less allergic/atopic [2–4].

Further, the SARP study found that the effect of obesity is different and more pronounced in early-onset asthma than in late-onset asthma [47]. Thus, asthma beginning in childhood, when being persistent, might be significantly complicated by obesity developing later in life whereas asthma beginning later in adulthood (possibly to the already obese patient) may not be so much more complicated by obesity as obesity may already have been a major driving factor in its appearance. In future, it is of utmost importance to differentiate between patients having asthma and becoming obese later and obese patients with new-onset asthma.

In a small Canadian study [46] (Tables 1(a) and 1(b)), the group of obese asthmatics presented with lower asthma control and had reduced total lung capacity (TLC), expiratory reserve volume (ERV), functional residual capacity (FRC), and residual volume (RV) compared to the normal weight asthmatics [46]. It is well-known that obesity is associated with a reduction in ERV and FRC [53–55], but it still remains contradictory, whether obesity or BMI as such is associated with more severe airway obstruction (FEV_1_/FVC) (see Tables 1(a) and 1(b)).

How existing obesity affects the therapy, control, and prognosis of asthma in a prospective setting? We identified one 12-month prospective disease management study that evaluated the impact of obesity in asthma [43]. In children, there was no relationship between BMI and severity of asthma, spirometry findings, quality of life (QoL), or healthcare utilization. In adults, there was no relationship between BMI and asthma severity or health care utilization but higher BMI was associated with a significant reduction in QoL and the BMI had an inverse relationship with forced vital capacity (FVC) [43]. The association of obesity with asthma control has been analysed in a large asthma population (Kaiser Permanente Southern California) (*n* = 10,233) [56]. Overweight and obesity were associated with increased relative risk for asthma hospitalizations or emergency department visits (RR 1.4). Obesity was associated with an increased risk for dispensing ≥ 7 short-acting beta-agonist canisters [56]. These analyses suggest that obesity may have different impact on asthma in younger age groups than in adults and that obesity is associated with more asthma symptoms, reduced TLC, and poorer asthma control.

### 3.3. The Effect of Weight Loss on Asthma and the Effect of Obesity on Pharmacotherapy

Does weight loss improve asthma control? A randomized study by Stenius-Aarniala and coworkers [57] showed that weight loss in 38 obese physician-diagnosed asthmatics is associated with improvements in asthma symptoms, lung function, and health status. This has been confirmed in another study [58] showing that dietary restriction, with or without exercise resulted in a greater benefit in terms of asthma control. Recently, a small controlled study (*n* = 16 + 6) reported that significant weight loss (from 115 kg → 98 kg/BMI 45 → 36.5) was associated with a significant improvement in PC_20_ (5 → 10 mg/mL), FVC% predicted (94 → 100), asthma control (ACQ score 1.4 → 0.6), and asthma quality of life (AQLQ score 5.6 → 6.1) [59]. In addition, small studies [60–62] have suggested that surgically induced weight reduction is associated with benefits in asthma control, symptoms, hyperreactivity, use of medication, and lung function. Markers of systemic inflammation (hsCRP, adiponectin, and leptin) were reported to be lower after bariatric surgery, whereas only a decrease in mast cells was reported in a group of asthmatics undergoing bariatric surgery [61]. However, the published studies are small (actively treated asthma patients together 27 + 23 + 12) [60–62] and the results suggest that the benefit may diminish over time [60]. Another factor that makes it difficult to assess the effect of weight loss interventions on asthma is that bariatric surgery improves lung function (e.g., FEV_1_, FRC, and TLC) in obese patients without asthma [61], suggesting that all improvement is not specific to asthma. A recent self-controlled case series study [63] of obese patients with asthma (*n* = 2261) and undergoing bariatric surgery gives hope that weight loss and bariatric surgery are associated with clear benefits in asthma. Significantly fewer emergency department (ED) visits or hospitalizations for asthma exacerbations occurred within 12 months after bariatric surgery (11% of patients) compared to during the reference period (22% of patients) and the risk remained low in the subsequent period of 13 to 24 months after bariatric surgery (11% of patients) [63]. In practical terms this means that before bariatric surgery 22 patients out of 100 experienced ED visit or hospitalization and after surgery only 11 out of 100 experience such adverse event because of asthma.

Obesity may also affect the response to the pharmacotherapy of asthma. The basic choices of pharmacotherapy [22, 31] of asthma as such are valid also in obese asthmatics. However, the obese asthmatics may be less responsive to standard asthma therapy with ICS as compared with normal weight patients [38] even though the findings are not consistent.

## 4. Metabolic Syndrome and Asthma

Obesity (high waist circumference) is a major component of metabolic syndrome, a cluster of metabolic components that indicate a significantly increased risk of cardiovascular disease [64]. The other components of metabolic syndrome include elevated triglycerides, reduced high-density lipoprotein (HDL) cholesterol, high blood pressure, and elevated glucose or diabetes. In contrast, the association of metabolic syndrome as a syndrome (i.e., not its single components) with asthma has been much less studied [64]. The Norwegian prospective cohort of the Nord-Trondelag Health Study from 1995 to 2008 [65] reported that metabolic syndrome was a risk factor for incident asthma (OR 1.6). Among components of metabolic syndrome, two remained associated with incident asthma: high waist circumference (OR 1.6) and elevated glucose or diabetes (OR 1.4) [65]. In the CARDIA study abdominal adiposity, elevated blood pressure, and impaired fasting glucose or diabetes were significantly associated with incident asthma. However, after adjustment for BMI their significance was lost suggesting that BMI as such is an independent risk factor for asthma in women [41]. In Korean 40–69-year-old adults, symptoms suggestive of asthma such as wheezing, resting dyspnea, and postexercise dyspnea were increased in the subjects of the metabolic syndrome group [66]. Abdominal obesity and hypertension were the risk factors for asthma-like symptoms among the components of the metabolic syndrome [66].

The association between obesity and asthma has been already largely studied (see above sections) and the association between DM2 and asthma will be dealt with separately in Section 5. Next, we will shortly review the associations between lipid metabolism and hypertension with asthma. The complex cholesterol and lipoprotein biology [67, 68] are outside the scope of the present review and the oversimplistic “good and bad cholesterol” view does not represent the whole phenomenon. Although several experimental animal studies as well as some epidemiological and clinical studies in children and adolescents [67, 68] suggest an association between cholesterol/lipoproteins and asthma, surprisingly few studies have been published on adults with asthma. Two early studies [69, 70] report conflicting findings in comparing the cholesterol/lipoprotein profiles in adult patients with asthma and healthy controls. A very recent study [71] evaluated the levels of apolipoprotein A-I and large high-density lipoprotein in adult atopic asthma. Serum levels of these positively correlated with FEV_1_, whereas serum triglycerides, LDL cholesterol, and apoB were associated with more severe airflow obstruction [71]. Even though the correlations were weak and only atopic asthmatics were included (possibly excluding a larger part of the nonatopic adult-onset asthma population) in this cross-sectional study, it raises an interesting possibility. It suggests that the atherogenic lipid profile is associated with reduced FEV_1_ in asthma [68] creating an important link between metabolic syndrome and asthma.

Data from Canadian Community Health Survey with self-reported or self-reported doctor-diagnosed conditions has shown in two different studies that patients with asthma were having approximately 1.4-fold risk for high blood pressure [72, 73]. Similarly, in a sample of Jackson Heart Study, use of medication for hypertension was more common among those African American women reporting current doctor-diagnosed asthma or use of medication to treat asthma [74]. Taking medication for hypertension was a significant risk factor for current asthma in this population [74]. A similar finding has been reported among Arab Americans [75]. Taken together, there is evidence from the epidemiological studies that hypertension and asthma are connected. However, there is scarcity of data from clinical studies whether hypertension complicates asthma or vice versa. A recent study using Kaiser Permanente Southern California registry data showed that hypertension is associated with markers of increased severity of asthma such as use of > 6 canisters of short-acting *β*_2_-agonist in a 12-month period, oral corticosteroid dispensing, and history of emergency department visits or hospitalizations [76].

Taken together, it still remains unclear whether metabolic syndrome itself is a risk factor for asthma or is the risk factor one (or several) of its components (obesity?). In addition, the impact of MBO or its therapy on clinical asthma remains mostly unknown [64].

## 5. Diabetes Mellitus and Asthma

Type I diabetes has been suggested to be an autoimmune disease due to environmental factors, possibly viruses, whereas type 2 diabetes mellitus has been connected with obesity, insulin resistance, and systemic inflammation [77–80].

An analysis of the large Kaiser Permanente Medical Care program has evaluated the risk of asthma among those with diabetes. In adults (aged ≥ 18 years) with no diabetes age- and sex-adjusted incidence of asthma was 0.16 per 1,000 person-years and in those with diabetes the corresponding figure was 0.41 indicating a higher risk for incident asthma [78]. This association between increased prevalence of asthma in patients with DM2 has also been confirmed in a large Danish twin study [79] and in hospitalized patients [80]. The proposed mechanisms how asthma could increase the risk for DM2 include genetic pleiotropy, lung-related inflammatory cytokines and their effects on insulin sensitivity, direct effects of hypoxia on glucose metabolism, and adverse early-life exposures and their effects on organ development [81]. In addition, severe asthma is treated with repeated courses or persistent per oral glucocorticoids that may increase the risk of type 2 diabetes [32] even though this has not been confirmed in other studies [82, 83]. Risk estimates for diabetes in patients with asthma have varied from 1.3 to 2.1 [81, 84, 85]. Asthma-diabetes association appeared stronger for adult- versus child-diagnosed asthma cases, and for participants who were obese compared to those who were nonobese [81]. The current data suggests that a connection between asthma and DM2 diabetes exists and suggests that both diseases are able to increase the risk of the other disease. In contrast, the effect of DM2 on asthma outcome remains unknown.

## 6. Cardiovascular Diseases and Asthma

Previously connection of asthma and arteriosclerosis has been evaluated in studies of asthma mortality or in studies among elderly patients with asthma. Patients with severe asthma have been reported to have a higher mortality from ischemic heart disease especially among women [86]. However, such a connection between these diseases has not been found in elderly asthma patients [69, 87].

### 6.1. Are Asthma and Cardiovascular Diseases Associated?

A large cross-sectional study (*n* = 16,943) looked for the associations between adult-onset asthma and CVDs including stroke, congestive heart failure, and coronary heart disease [88]. Adult-onset asthma (age of onset ≥ 18 years) was found to have a significant association with total CVD (OR 2.1). Only coronary heart disease (CHD) was significantly associated with adult-onset asthma (OR 2.3). In gender stratified analyses, only females with adult-onset asthma showed an increase in the odds for total CVD (OR 2.4) and CHD (OR 2.9). In contrast to expectations, overweight (BMI 25–30) females with adult-onset asthma had stronger association with CVD than those having adult-onset asthma and being obese (BMI > 30) [88]. The limitations of the study were the cross-sectional setting and definition of smoking history (nonsmokers or current smokers) thus making exclusion of COPD impossible [88].

### 6.2. Does Asthma Increase the Risk of Cardiovascular Diseases?

Over the past decade large cohort studies have been published to assess the risk of cardiovascular events as asthma comorbidities. In a large cohort study of health insurance database (*n* = 151,620) connection of asthma and combined nonfatal or fatal CHD was assessed [89]. Patients (age ≥ 18 years) with self-reported physician-diagnosed asthma or hospitalization due to asthma were followed for 27 years. In women, the age-adjusted CHD rate was higher in those with asthma than in those without asthma with a HR of 1.2. Asthma was associated with the risk of developing CHD in both younger (<50 years) (HR 1.2) and older (≥50 years) women (HR 1.2). Asthma among women became significantly associated with CHD events already after 10 years of follow-up (HR 1.2). This suggests that women might have a greater biological susceptibility to the inflammatory asthma milieu or to the cardiotoxic or metabolic effects of asthma medications [89].

Several studies of the Arteriosclerosis Risk in Communities (ARIC) cohort have been published concerning asthma and CVDs as comorbidity [90]. In a prospective study with 14 years of follow-up asthma was modestly associated with an increased incidence of stroke [91]. In a further study of the same cohort [92] evaluating the relationship between asthma and carotid artery intima-media thickness (IMT) also the age at asthma onset was taken into consideration [92]. The weighted mean for wall IMT thickness for women with history of adult-onset asthma (≥21 years) was significantly greater than that of women without asthma. In contrast, IMT in women with a history of childhood-onset (<21 years) asthma did not differ substantially from nonasthmatic women. Third study of the ARIC cohort [93] evaluated incidence of CHD and stroke among asthma subtypes (asthma onset < or ≥ 21 years). Women, but not men, with adult-onset asthma were found to have a 2-fold increased rate of CHD compared to their nonasthmatic counterparts.

Iribarren and coworkers [94] followed two matched adult cohorts, the other with asthma and a parallel asthma-free cohort. Both cohorts were followed up for 12 years for incident nonfatal or fatal CVD and all-cause mortality. Asthma was associated with a 1.4-fold increased risk of coronary heart disease, a 1.2-fold risk of cerebrovascular disease, a 2.1-fold risk of heart failure, and a 3.3-fold risk for all-cause mortality. Similarly to that found in the ARIC cohort [92, 93] stronger associations were noted among women, but there was no data on asthma onset or asthma severity [94]. Among patients with asthma, 84% used one or more asthma medication and particularly those using oral corticosteroids alone or in any combination with asthma medications were at enhanced risk for developing CVD, which may reflect that that they were having severe asthma [94]. There remains a possibility that the medication used may increase the risk of CVD as especially *β*_2_ agonists have many adverse cardiovascular side-effects and continuous use of glucocorticoids may have many metabolic disadvantages [89, 94].

Recently, a long-term (10 years) prospective study reported an association of persistent asthma and CVD events (coronary death, myocardial infarction, angina, stroke, and CVD death) [95]. All participants (*n* = 6792) were free of CVD at baseline. Self-reported asthma cases (*n* = 667) were classified as persistent (daily use of controller medication such as ICS, LTRA, and OS) in 77% and intermittent (without controller medication) in 23% of patients. After a decade of follow-up patients with persistent asthma had a 1.6–1.7-fold higher risk of CVD events than nonasthmatics. In addition, markers of systemic inflammation (IL-6, CRP, alfa-dimer, and fibrinogen) were the highest in patients with permanent asthma even though patients were using modern controller asthma medication. Use of controller medication did not abolish the increased risk of CVD events [95].

Current and former smoking are one of the main risk factors for both asthma and cardiovascular diseases. Recently published report [96] from the Copenhagen General Population study cohort (*n* = 94,079) assessed prospectively the risk of asthma and CVD including ischemic heart disease, myocardial infarction, and ischemic stroke among smokers and nonsmokers. Six percent (*n* = 5691) had self-reported asthma, 40% were never smokers, 43% former smokers, and 16% current smokers. Mean follow-up time of the study patients (age 20–100 years) was 4.5 years. Hazard ratios among individuals with asthma compared to never smokers without asthma for ischemic heart disease were 1.5 in former smokers and 2.0 in current smokers. The respective corresponding values were 1.7 and 3.2 for myocardial infarction and 1.2 (n.s.) and 3.0 for ischemic stroke. In contrast to previous studies the increased risk for cardiovascular comorbidities in this cohort study was restricted only to smokers with asthma [96]. Unfortunately data on the age at asthma onset was not available as in other cohorts the increased risk of CVD was seen mostly only in women with adult-onset asthma. In addition, the follow-up time (4.5 years) is relatively short for the never smoker asthma group as they were women with a mean age of 53. The risk of myocardial infarction in smoking persons increases more early but in never smokers (especially women) it clearly increases only after the age of 60 [97]. In most asthma comorbidity studies confounding effect of smoking has been taken into account. Usually patients have been divided into groups of current, ever, and never smokers. In some studies data on pack-years is available. However, more detailed information like duration, intensity, and type of smoking as well as age of smoking onset and second hand smoking should be part of the multivariate models especially in studies concerning asthma and the risk of cardiovascular diseases [98].

These cross-sectional and cohort studies with large populations have shown that asthma patients and especially a subgroup of adult-onset asthma women are at increased risk of arteriosclerosis. In future, it would be important to know whether totally controlled asthma would reduce the risk of cardiovascular comorbidities. The role of contemporary asthma medication to prevent or to enhance incidence of cardiovascular diseases also remains obscure. Furthermore, we lack knowledge of how different CVDs affect asthma outcome and whether CVDs are of importance in making decisions on how to modify treatment of asthma.

## 7. Mental Disorders, Suicide, and Asthma

The association between asthma and psychological factors has been recognized for centuries [99]. Psychosocial factors have long been suspected to influence the onset and course of asthma. These effects may be mediated via direct influences such as influences on the pulmonary system, autonomic and endocrine regulation, and inflammatory and immune process (see later in this paper and [100]). In addition, psychosocial factors can influence asthma via multiple indirect pathways such as medication adherence, perception of airway obstruction (either overperception or underperception), illness beliefs, or general health behavior [100].

A considerable number of studies have suggested that there is an association between some mental disorders and asthma, especially severe asthma [99–101]. Studies conducted among clinical and general practice samples have found higher than expected rates of anxiety disorders (particularly panic disorder) and of depression among adult patients with asthma [99]. However, the published results have not always been consistent and methodological problems (e.g., not using DSM-based diagnostics and variability in the assessment of asthma) make it difficult to draw clear conclusions [99, 100].

### 7.1. Is There Association between Asthma and Mental Disorders?

The World Mental Health Survey performed in 17 countries (*n* = 85,088) using general population sample reports that an adult with asthma has an increased risk for depressive disorders (OR 1.6) and anxiety disorder (OR 1.5) as compared with persons not having asthma [101]. A meta-analysis estimating the prevalence of anxiety disorders in asthma reported that the average prevalence of any anxiety disorder among adults with asthma was 34% [102]. More specifically, the prevalence of panic attacks (25%), panic disorder (12%), agoraphobia (12%), and generalized anxiety disorder (9%) was higher among adults with asthma than in the general population [102]. A recent meta-analysis has evaluated the association between asthma and depression [103]. Patients with depression had increased risk for having asthma (OR 3.2) as compared with those not having depression and patients with asthma had an increased risk for having depression (OR 1.5) as compared with those not having asthma [103]. A similar association has been reported from Spanish National Health Survey [104] reporting that asthmatic patients suffer more often from anxiety (9.7%) and depression (9%) than persons not having asthma (6.6% and 5.5% for anxiety and depression, resp.). Having asthma increased the probability of suffering from anxiety (OR 1.3) and depression (OR 1.4). Among asthmatics factors associated with suffering from anxiety were older age, concomitant comorbidities, and visits to GP practices in the last 4 weeks, whereas factors associated with depression were female sex, older age, worse self-related health, concomitant comorbidities, abstemious individuals, and the need for attendance on emergency room in the last year [104]. A similar association has been reported also from Germany reporting that lifetime severe asthma was significantly associated with an increased risk for a number of anxiety disorders (any anxiety disorder, panic disorder, panic attacks, social phobia, specific phobia, and generalized anxiety disorder), bipolar disorder, and any severe mental disorder [105]. Lifetime nonsevere asthma was significantly associated with having any anxiety disorder, not specified anxiety disorder, and with any somatoform disorder [105]. It appears that the association between anxiety/depression and asthma is not limited to Western countries as similar association has been reported from Chinese population [106] and from an analysis of self-reported data from most of the evaluated 54 countries [107]. In addition, the association between asthma and/or atopic diseases with anxiety and depression is not limited to adults only but has been reported also in children [108] and adolescents [109].

In a Norwegian population-based survey [110] cases with a personality problem evaluated using the Iowa Personality Disorder Screen more frequently reported asthma along with other chronic conditions such as persistent muscular pain, fibromyalgia, and alcohol problems. Asthma (ever suffered from asthma) was found to be of borderline significance in influencing the prevalence of personality problems [110]. As patients with personality problems have increased somatic morbidity and primary health care utilization these results raise another possible cluster of diseases, namely, association between asthma and personality problems and persistent musculoskeletal pain and fibromyalgia that are generally considered as “noninflammatory” diseases. A possible link between these conditions and asthma may be the increased use of nonprescribed analgesics by these patients.

Suicide is a major public health issue worldwide often associated with mental illnesses. Asthma can have a significant effect on quality of life, and consequences include sleeplessness, daytime fatigue, reduced activity levels, school and work absenteeism, and mental health problems [111]. Thus, it can be hypothesized that asthma could be associated with suicide-related behavior. A recent review [111] collected the available observational studies between asthma and suicide-related behavior (ideation, attempts, and completion). The evidence from six different observational studies support an association between asthma and suicide-related behavior the risk being 1.4–3.5 times higher in patients with asthma as compared with nonasthmatic counterparts [111].

### 7.2. Does Asthma Predispose to Mental Disorders?

The only study identified to address this connection evaluated whether asthma predisposes to mental disorders in a longitudinal setting in a Danish birth cohort (children born between 1977 and 1992 and followed up from the age of 15 until year 2008) when they were evaluated for the presence of schizophrenia [112]. Asthma was associated with an increased risk (RR 1.6) for developing schizophrenia and the risk was confirmed in a case/sibling study [112]. Due to its setting (free of charge Danish public health care), this study most probably avoids most of the biases usually related to studies on mental disorders. However, it evaluates only hospital discharge and outpatient data and may thus exclude the less severe cases of asthma.

### 7.3. Do Mental Disorders Predispose to Asthma?

As a part of World Mental Health surveys (*n* = 18,303) the association between childhood adversities (physical abuse, sexual abuse, neglect, parental death, parents divorce, another parental loss, parental mental disorder, parental substance use, parental criminal behavior, family violence, and family economic adversity) and early-onset depressive and anxiety disorders and adult-onset asthma has been evaluated [113]. Childhood adversities predicted adult-onset asthma with risk increasing with the number of adversities experienced (HRs 1.5–1.7). However, also early-onset depressive and anxiety disorders independently predicted adult-onset asthma (HRs 1.7–2.1). This suggests that mental disorder-asthma relationship is independent and not a function of a shared background of childhood adversity [113]. Data collected from face-to-face household surveys in community-dwelling adults (*n* = 52,095) from 19 countries evaluated the lifetime prevalence and age at onset of 16 DSM-IV mental disorders as well as self-reported physician diagnosis of asthma and age at onset. After adjustment for comorbid mental disorders several mental disorders were associated with subsequent adult asthma onset: bipolar disorder (OR 1.8), panic (OR 1.4), generalized anxiety (OR 1.3), specific phobia (OR 1.3), posttraumatic stress (OR 1.5), binge eating (OR 1.8), and alcohol abuse (OR 1.5). Mental comorbidity linearly increased the association with adult asthma. The association with subsequent asthma was stronger for mental disorders with early onset (before age of 21) [114]. The major biases in these multinational analyses may relate to cultural differences in the diagnostic accuracy when questions on doctor-diagnosed asthma are presented or the World Mental Health survey tools are used.

### 7.4. Do the Mental Disorders Affect Clinical Asthma?

Anxiety and other psychiatric morbidities are often advised to be considered in differential diagnostic and when estimating treatment options in severe asthma or asthma not responding to treatment. Despite this common advice there exist relatively few studies that have directly evaluated how the mental disorders affect clinical asthma. The effects of comorbid mental disorders on health care service use and psychosocial factors in asthma have been reviewed recently [100, 115]. In this review we included only studies more recently published or most relevant to adult asthma. A clinical sample of adult Canadian patients with asthma (*n* = 504, not specifically adult-onset asthma) was subjected to evaluation of the presence of depressive and/or anxiety disorders by using the Primary Care Evaluation of Mental Disorders Interview [116]. Almost one-third of the patients fulfilled the diagnostic criteria for either condition (8% were depressive only and 12% had anxiety only) or both (11%) of these disorders. Depressive disorder had an independent worsening effect on asthma control, whereas both depressive and anxiety disorder independently impaired asthma-related quality of life [116]. In contrast, there were no significant differences in other asthma-related endpoints (lung function, severity, atopy, emergency department visits, hospitalization in the last year, or asthma medication) [116]. The same research group has more recently analysed the impact of generalized anxiety disorder on asthma morbidity in a sample of adult patients with confirmed asthma (*n* = 749, not specifically adult-onset asthma) [107]. Generalized anxiety disorder affected 4% of the patients and impaired asthma control and asthma-related quality of life, increased bronchodilator use, and was associated with worse asthma self-efficacy [117]. However, the comorbid major depressive disorder and low asthma self-efficacy may account for many of these associations [117]. In contrast, there was no association between generalized anxiety disorder and emergency visits or hospitalizations [117]. This is in line with a systematic review made on psychiatric morbidity and asthma and health care costs [115] in which mood disorders (depression), posttraumatic stress disorder, and not specified mental disorder were found to increase the risk for emergency department visits (ORs 2.6, 5.2, and 4.6, resp.). In contrast, results for anxiety/panic disorder were not consistent between studies [115], although they suggest a trend towards increased emergency department visits. Similarly, increased rate of general practitioner visits was found with patients having depression, posttraumatic stress disorder, and not specified mental disorder but the results with anxiety/panic disorder were not consistent [115]. A recent analysis evaluated the effect of panic disorder and panic-anxiety on asthma control, health service use, and quality of life in adult patients with asthma (*n* = 643, not specifically adult-onset asthma) during a 4-year follow-up [118]. Panic disorder and anxiety sensitivity index (ASI) scores predicted worse follow-up ACQ total scores but not AQLQ scores [118]. ASI scores also predicted greater nocturnal and waking symptoms, activity limitations, and bronchodilator use. In contrast, neither panic disorder nor ASI scores were associated with hospitalisation, although ASI score was marginally associated with an increased risk of emergency department visits (RR 1.023) [118]. Among severe asthmatics the presence of a psychiatric disorder has been reported to increase the risk for frequent visits to GP (OR 5.9), frequent emergency visits (OR 5.3), frequent exacerbations (OR 12.4), and frequent hospitalizations (OR 4.8) even though these patients do not differ from those not presenting with psychiatric disorder in demographic or objective disease characteristics [119]. There exists a possibility that the deleterious effects of mood or anxiety disorders on asthma control might be mediated via behavioural effects such as tobacco smoking. A recent study [120] evaluated this and found that current smoking, having an anxiety disorder, and having a mood disorder are independently associated with poorer asthma control. Recently, it has been proposed that the depressive symptoms mediate the relationship between BMI and worse asthma control [121] although this has not been confirmed in another study [122].

Mental disorders are often associated with increased rate of work absence. The results of two separate studies [35, 123] suggest an increased rate of work absence in asthma with comorbid depression (ORs 3.6–4.0). In a Finnish analysis of public sector employees [35], the risk was especially high for permanent work disability (HR 6.8) [35].

There remain a number of possible problems with the studies evaluating the effects of comorbid mental disorders on the outcome of clinical asthma. Firstly, many studies are cross-sectional and long-term follow-up studies are lacking. Secondly, many studies rely on self-reported data rather than clinical assessment or collection of clinically proven events from patient records. Thirdly, the patient population in clinical studies often represents tertiary care; that is, the group of patients with asthma is highly selected. Fourthly, smoking and differential diagnostics towards COPD are not taken into account. However, there exists enough evidence to suggest that psychiatric comorbidities associate with asthma and may negatively affect its outcome. Thus, long-term follow-up studies evaluating the effects of different mental comorbidities on asthma outcome are needed.

## 8. Mechanisms Linking Asthma with Its Comorbidities

Considering the high coexistence of obesity, metabolic syndrome, type 2 diabetes, and depression with asthma, it is not surprising to find many overlapping pathways in their possible pathogenetic mechanisms. Other than traditional, Th2-related mechanisms are likely to explain the coexistence, because the current reports suggest no clear association between BMI and eosinophilic airway inflammation in asthma. To briefly review studies concerning airway inflammation in obese asthma, no difference was found in airway inflammation in morbidly obese subjects with or without asthma [124]. Increase in bronchial submucosal eosinophils, even though not in blood or sputum eosinophils, was reported in severe obese asthmatics when compared to leaner asthmatics [125]. Inverse or no relationship between BMI and sputum/blood eosinophilia or FeNO has commonly been described in patients with asthma. Furthermore, no consistent results exist on predominating neutrophilic airway inflammation either [46, 126–128]. Thereby, it is likely that the increased symptoms in obese patients with asthma are not explained by increased airway inflammation. Next, we will continue by describing the mechanisms that link the pathogenesis of comorbid conditions such as obesity, type 2 diabetes, cardiovascular diseases, and depression with asthma. Due to high degree of overlapping mechanisms, the section is sectioned by mechanisms instead of separate comorbid conditions.

### 8.1. Early Life Exposures

Early life exposures such as maternal diet, maternal smoking, air pollution, and pregnancy complications affect metabolic health of the offspring and the risk of developing chronic disease later in life. For example, maternal obesity has been associated with both increased risk of insulin resistance and asthma of the offspring during childhood [129, 130]. Also low birthweight has been associated with increased risk of having asthma, hypertension, diabetes, or heart diseases by age of 50 years [131]. These risks may be mediated by structural, metabolic, or epigenetic changes that occur in foetus in response to adverse environment in utero [132]. For example, epigenetic modifications such as DNA or histone methylation and histone acetylation in foetal genes important for lipid or carbohydrate metabolism or immune system are likely mechanisms involved in explaining later risk and higher occurrence of many metabolic and other chronic diseases [133].

Severe early life stress has been associated with depression as well as asthma later in life [134–137]. To explain this association, severe childhood stress was shown to predispose to persistently high inflammatory status of the body (CRP and IL-6) lasting to adulthood. In subjects with severe childhood adverse events the systemic levels of inflammatory cytokines were also raised higher in response to an adverse event in adulthood when compared to those without childhood adverse events [134, 135]. The persistently activated inflammatory pathways may increase susceptibility to other chronic inflammatory diseases.

### 8.2. Systemic Inflammation

Systemic inflammation is a phenomenon common for obesity, diabetes mellitus, depression, and at least certain phenotypes of asthma constituting a possible link between these disorders [103, 138]. Systemic inflammation is generated by adipose tissue at the obese state or can be developed, for example, in response to severe social stress. It is characterized by increased levels of inflammatory markers such as C-reactive protein (CRP), interleukin- (IL-) 6, IL-1*β*, tumor necrosis factor- (TNF-) *α*, and leptin. Elevated IL-6, CRP, and soluble CD163 (macrophage activation marker) have been associated with reduced lung function (especially in severe asthma) and neutrophilic airway inflammation [138–140]. TNF-*α* has also been shown to enhance eosinophil survival [141]. IL-6 possesses wide effects on innate immune system and has been proposed to play an active role in asthma pathogenesis. IL-6 may affect asthma development, for example, by stimulating neutrophil recruitment and T helper cell differentiation into Th2 or Th17 cells (the latter in the presence of TGF-*β*) or by stimulating IL-13 production by T helper cells [142, 143]. Th17 cells are involved in neutrophil recruitment; they are steroid-resistant (in contrast to, e.g., Th2 cells) and considered to be significant players in severe neutrophilic asthma. Recently, higher amount of dual-positive Th2/Th17 cells was found from BAL of patients with refractory asthma when compared to disease control subjects [144]. IL-6 and IL-1*β* are cytokines able to induce differentiation of Th2 cells into Th2/Th17 cells and are likely involved. Amount of Th2/Th17 cells as well as IL-17 levels in these patients positively correlated with airway hyperreactivity and eosinophil counts and negatively correlated with FEV_1_ suggesting that Th2/Th17 cells associate with severe asthma. Moreover, dual-positive Th2/Th17 cells were resistant to proapoptotic effects of glucocorticoids [144]. Given that obese patients with asthma are less sensitive to treatment with steroids [38] either Th2/Th17 or Th17 induction by IL-6 may play a significant role in the disease process. Because obesity stimulates systemic inflammation, and cytokines of systemic inflammation skew Th cells into Th17, elevated levels of Th17 cells and elevated production of IL-17 would also be expected in obese subjects and patients with type 2 diabetes. Indeed, this was shown in peripheral blood cells from patients with type 2 diabetes [145]. Moreover, 3–10-fold increases in Th17 cells were demonstrated in adipose tissue from obese patients with insulin resistance, when compared to obese insulin-sensitive or lean patients [146]. Taken together, Th17 cells may be induced in the milieu of systemic inflammation and play a role in pathogenesis of both insulin resistance and asthma. Th17 cells have also been shown to increase with age in healthy humans [147], and an experimental study suggested that development of Th2/Th17 milieu following allergic response would be related to older age [147, 148].

A recent study on gene expression of blood CD4+ T cells from patients with depression and asthma showed that the main active pathways in depressive asthma are those related to acute phase protein signalling (such as IL-6 and CRP signalling) [149]. Chronic stress may lead to dysregulation of hypothalamic-pituitary-adrenal (HPA) axis, long-term increase in the levels of cortisol, glucocorticoid resistance, and systemic inflammation. Cytokines elevated during systemic inflammation are able to generate symptoms such as fatigue and loss of appetite that overlap with symptoms of depression [137, 150]. Moreover, reduced serotonin neurotransmission is present in depression and inflammatory cytokines elevate enzyme indoleamine-2,3-dioxygenase (IDO), which degrades the most important precursor of serotonin, tryptophan [151].

### 8.3. Inflammasome

Nod-like receptor protein 3 (NLRP3) inflammasome is a multiprotein complex at the interface of metabolism and inflammation and involved in the development of systemic inflammation. Its activation is a two-step process leading to release of IL-1*β* and IL-18, mainly in the cells of innate immunity. The interest lies in its ability to be activated by wide array of dangerous substances ranging from pathogens to metabolic compounds (elevated extracellular glucose, amyloid-*β* peptide, and oxidized low-density lipoprotein (LDL)), extracellular acidosis, and number of environmental irritants [152]. Also psychological stressors have been shown to elevate IL-1*β* and may therefore activate inflammasome, even though direct activation mechanisms have not been demonstrated [153]. The precise mechanisms of NLRP3 inflammasome activation by these various factors are largely unknown. Extensive literature supports the role of ROS and mitochondrial dysfunction in inflammasome activation, both being common in obesity, diabetes, and asthma [152, 154]. Studies with knock-out mice have suggested that under normal circumstances IL-1*β* is important for maintenance of adipose tissue homeostasis and has partially overlapping functions with IL-6. However, during inflammatory conditions and chronic stimulation, it drives obesity-related disease progression, for example, by interfering with insulin signalling in adipose tissue and liver. Interestingly, in mice IL-1*β* and TNF-*α* also upregulated serotonin transporter (SERT) gene that is central in inducing despair-like behaviour by uptake of serotonin to the presynaptic neuron [155, 156]. By this mechanism inflammasome and IL-1*β* could contribute to depression. Moreover, NLRP3 inflammasome has been found to be involved in ageing-related inflammation and cognitive decline [152].

Finally, development of airway hyperresponsiveness in response to high-fat diet in mice was shown to be dependent on NLRP3 inflammasome-mediated production of IL-1*β* and IL-17 [157]. IL-1*β* expanded IL-17A-producing type 3 innate lymphoid cells (ILC3s) in the lungs. Also patients with severe asthma were shown to have higher numbers of IL-17A-positive ILC3-like cells in BAL when compared to healthy subjects or patients with mild asthma [157]. Altogether, existence of wide array of activators of NLRP3 inflammasome suggests that it may be involved in translating metabolic or inflammatory danger signals into metabolic diseases, airway hyperresponsiveness, and even depression.

### 8.4. Adipokines

Adipokines have been widely studied in the context of finding the link between obesity and asthma. Adipokines are group of cytokines produced by adipose tissue and contain mediators with proinflammatory (e.g., leptin and resistin) and anti-inflammatory (e.g., adiponectin) functions. Serum levels of leptin increase with increasing BMI [158, 159] and are higher in females [160]. Both clinical and experimental evidence suggest that leptin functions by augmenting airway hyperresponsiveness but not by affecting inflammation [161–163]. In obese patients with adult-onset asthma, markers of metabolic inflammation, macrophage number, and leptin levels were increased in visceral fat when compared to obese controls and airway reactivity was significantly correlated to visceral fat leptin expression [161]. In nonobese women with adult-onset asthma, leptin correlated negatively with lung function and positively with asthma symptom score, after adjusting for BMI [164]. How metabolic inflammation in visceral fat and the mediators involved translates into airway hyperresponsiveness remains unknown. Adipokine receptors are expressed in the airway epithelium and smooth muscle cells and in eosinophils suggesting that there may be direct effects on the structural and inflammatory cells of the airways [161, 165, 166]. In eosinophils, leptin has been shown to promote survival [167, 168].

Adiponectin is an adipokine with insulin-sensitizing and anti-inflammatory effects, and low level of adiponectin has been associated with both incident asthma and metabolic disorders, even though the association has not been consistent [169, 170]. Currently, the primary target cells that are important for the beneficial anti-inflammatory effects of adiponectin are unknown. Adiponectin circulates in the blood only in multimeric forms with low, medium, or high molecular weights and their distribution differs in men and women [171]. Indeed, quite differential associations have been found between adiponectin levels and metabolic diseases or asthma in men and women, and the mechanisms may be largely gender-specific [172, 173]. Even though adiponectin has been demonstrated to suppress activation of many proinflammatory molecules in adipocytes and macrophages in previous studies [174, 175], very recently the effect of adiponectin on inflammation was shown to be dependent on macrophage phenotype. In classically activated mouse M1 macrophages adiponectin increased levels of proinflammatory cytokines IL-6, TNF-*α*, and IL-12 but in alternatively activated M2 macrophages it enhanced production of anti-inflammatory IL-10 [176]. Even though M2 macrophages are prevalent in traditional allergic asthma, obesity is associated with increase in M1 macrophages at least in adipose tissue [177], and thereby adiponectin might actually exert proinflammatory effects in obesity-associated conditions. More studies are needed to clarify the issue.

### 8.5. Hyperglycemia and Hyperinsulinemia

Studies have shown 3–10% lower lung function (more consistently FVC than FEV_1_) in adults with diabetes (without asthma) when compared to adults without diabetes and the association was independent from obesity or smoking. In longitudinal studies, reduced baseline lung function (mostly FVC) was also shown to predict incident diabetes [178]. Hyperglycemia and hyperinsulinemia follow insulin resistance and are both prevalent in metabolic syndrome and, in untreated type 2 diabetes, hyperinsulinemia is also present in treated type 2 diabetes. The effects of high glucose or insulin on the lungs have received interest in attempts trying to find an explanation to the association between diabetes and reduced lung function.

Experimental studies and development of inhalation formula of insulin have provided some interesting insights to the direct effects of glucose and insulin on the lungs. In experimental studies, insulin has been shown to contribute to formation of hypercontractile airway smooth muscle cells by promoting expression of laminin [179]. To support this, inhalation of insulin by diabetic patients resulted in cough and decrease in FEV_1_ [180]. Insulin also promotes fibroblast proliferation and differentiation resulting in collagen deposition, thereby contributing to airway remodelling [181, 182]. However, infusion of insulin into obese subjects led to anti-inflammatory effects in their peripheral blood mononuclear cells. Insulin reduced short-term activation of NF-*κ*B and expression of many inflammatory mediators (IL-4, ADAM-33, LIGHT, and LTBR) related to asthma in these cells [183, 184]. In experimental studies, insulin has also contributed to Th2 response and induced mast cell survival and degranulation [181, 182].* In vitro*, high glucose concentration was shown to enhance responsiveness of airway smooth muscle cells to contractile agents and to enhance intracellular calcium release [185]. Altogether, chronically high insulin or glucose levels in the lungs may promote airway hyperresponsiveness and airway remodelling and thereby contribute to reduced lung function or incident asthma in patients with diabetes. Other mechanisms proposing to explain the reduced lung function in patients with diabetes are microangiopathy of the alveolar capillaries and pulmonary arterioles, as well as autonomic neuropathy [178, 186].

Hyperglycemic condition is common in patients with acute asthma exacerbations, and generally disturbance in glucose metabolism has been described in patients with asthma, even in the absence of any diabetic risk factors [187, 188]. Hyperglycemic condition may result from treatment with systemic steroids and/or *β*_2_-agonists [187, 189]. Glucocorticoids promote gluconeogenesis in liver and antagonize insulin response in skeletal muscle and adipose tissue leading to hyperglycemia. However, treatment with inhaled steroids seems not to predispose for insulin resistance or diabetes [190].

### 8.6. Lung Mechanics

Obesity underlies many chronic conditions and its direct effects on lung mechanics may also contribute to development of asthma in these conditions. In obese subjects, the increased soft tissue compresses the thoracic cage leading to higher frequency of breathing, reduced peripheral airway diameter, and reduced lung volumes, which may lead to dyspnoea [37]. Recently it was shown that obesity is associated with increased resistance and dysfunction in peripheral airways [191]. However, something must exist to separate those obese subjects who develop asthma from those who do not. A weight loss study in obese nonallergic female asthmatics suggested that peripheral lung of the obese patients developing asthma may be more collapsible when compared to obese controls [192]. Natural variation in the airway wall stiffness and thickness could provide explanation for the collapsibility and explain why some obese individuals are prone to develop hyperresponsiveness and asthma, and others are not. The airway wall properties could be determined by genetics or be a result of obesity-related altered metabolism [193].

### 8.7. Mitochondrial Dysfunction

Mitochondrial dysfunction has been suggested as a common pathogenetic mechanism of obesity, metabolic syndrome, and asthma [194]. Mitochondrial dysfunction and higher level of oxidative damage in mitochondria has also been described in patients with major depressive disorder (MDD) [195, 196]. In obese subjects, caloric excess overloads mitochondria, leading to overproduction of reactive oxygen species, damage to mitochondrial (and cellular) components, and mitochondrial loss. Mitochondrial dysfunction results in incomplete oxidation of fatty acids and increased circulating lipids. Some metabolically active lipids inhibit insulin signalling and may promote insulin resistance. The situation is aggravated by physical inactivity, since physical activity is a way of increasing mitochondrial activity [194, 197]. Similar changes in mitochondrial function occur in older age [198]. Mitochondrial dysfunction has also been shown in experimental allergic asthma in mice [199, 200] but evidence in human asthma is limited to genetic studies [194]. Asthma is, however, associated with oxidative stress [201], which may be partially derived from dysfunction of mitochondria but also accelerates mitochondrial dysfunction.

### 8.8. Disturbed Nitric Oxide Metabolism

Disturbed nitric oxide metabolism is also common for asthma and metabolic disorders. Obesity, metabolic syndrome, depression, cardiovascular diseases, and asthma have all been associated with increased level of asymmetric dimethyl arginine (ADMA) which is an inhibitor of all nitric oxide synthase (NOS) isoforms [202–206]. In obese patients with asthma, decreased ratio of L-arginine/ADMA in plasma and its association with worse outcome of asthma were shown [207]. Endothelial NOS is critical for vasodilatation, and its inhibition by ADMA may also lead to endothelial dysfunction, increased risk for atherosclerosis, and other cardiovascular diseases [208]. In airway diseases, the adverse effect of ADMA is thought to be mediated by enhancement of oxidative stress, which was shown in murine lung epithelial cells and in allergically inflamed lungs of mice after administration of ADMA [209, 210]. Increased ADMA may direct NOS to form superoxide instead of NO and lead to oxidative stress, mitochondrial damage, and dysfunction. Mitochondrial dysfunction in turn contributes to insulin resistance and type 2 diabetes by the route described above. The NOS enzyme(s) most critical for these effects remain to be determined. Another mechanism that may affect development of asthma in response to NOS inhibition is increased availability of L-arginine, the starting substance of NO to arginase-1/arginase-2 resulting in increased production of polyamines and proline. Polyamines function by increasing airway hyperresponsiveness and eosinophil survival and proline is precursor of collagen, important for airway remodelling [211, 212]. Upregulated arginase-1 has also been demonstrated in patients with diabetes and in patients with heart failure [213, 214], postulating another common disease mechanism with asthma.

### 8.9. Leukotrienes

Leukotrienes construct another link between asthma and cardiovascular diseases. In asthma, cysteinyl leukotrienes (LTC4, LTD4, and LTE4) are well-known mediators of bronchoconstriction, mucus production, and inflammatory cell recruitment in the airways and produced mainly by eosinophils and mast cells. Leukotrienes are derived from activation of 5-lipoxygenase (5-LO). 5-LO is upregulated also in atherosclerotic lesions and the increased number of 5-LO+ cells was described during progression of coronary heart disease [215]. Additionally, higher count of 5-LO+ cells was found in atherosclerotic plaque samples from symptomatic versus asymptomatic patients with internal carotid artery stenosis. Plaque samples from these symptomatic patients showed also higher levels of LTB4 and active matrix metalloproteinases (MMP) 2 and 9 [216]. MMPs contribute to myocardial infarction by rupturing atherosclerotic plaques. Male hormones showed suppressive effects on 5-LO products [217] whereas estradiol stimulated release of cysteinyl leukotriene C_4_ from mast cells* in vitro* [218]. These hormonal influences might partly explain the stronger association between cardiovascular diseases and asthma in women when compared to men.

## 9. Conclusions and Future Perspectives

Studies concentrating on evaluating the risk factors of asthma, impact on clinical disease, or impact on prognosis give clearly different benefits for the clinician. If a comorbid condition is a risk factor for asthma, it means that a person having the comorbid condition (e.g., being obese) but not having asthma has a higher probability of becoming asthmatic at some point later in his/her life. This data can help us guide, for example, the obese patient to lose weight and thereby reduce the future risk of becoming asthmatic. In addition, this knowledge can help us to target the preventive measures at population level to reduce the risk of developing a certain disease. However, to help the clinician to decide how to treat the patient we need to know what is the significance of the particular comorbidity for asthma control, severity, and lung function and how it affects therapy. To understand this we need clinical studies that evaluate the effect of the comorbidity on asthma disease activity and outcomes in asthma.

The mechanisms dealt in this review mainly originate from patient and experimental data measuring the different pathophysiological events, cells, and cell functions and possible mediators that might be relevant in the interaction between the particular comorbidity and asthma. Thus, these mechanisms rather represent the situation where the comorbid condition is affecting the clinical outcome of asthma. Instead, they may not prove that a certain mechanism or mediator is causative or mediating the development of asthma. That kind of study would require a different experimental setting. For example, measurement of certain mediators in large population samples and following whether these people later develop incident asthma or not might help to resolve those questions.

For comorbidities such as MBO or its components, DM2 and CVD, we lack studies that evaluate their effect on clinical asthma. There exists some preliminary evidence on the deleterious effects of obesity and mental disorders on asthma outcome (Figure 1). Clinical studies that evaluate long-term prognosis and the contribution of obesity (or other comorbidities) on asthma are needed. In addition, studies that evaluate changes in the comorbidity over time on asthma control, exacerbations, lung function, and drug usage are lacking. Obesity has been in focus during last years. However, obesity itself is associated with significant comorbidity (such as diabetes, cardiovascular diseases, and mental diseases) and lifestyle factors (such as smoking, unhealthy diet, chronic alcohol use, and low level of physical exercise) that may contribute to the asthma outcome. From the mechanistic point of view it may be rational to include patients with only single comorbidity (e.g., obesity) to asthma studies at the time (see, e.g., Table 1(a)). However, from the perspective of practicing respiratory specialist or general physician this is not optimal as most patients suffer from multimorbidity rather than from a single disease or a combination of two specific single diseases. For example, in Germany most patients (approximately 60%) have one or more diseases out of eight listed in addition to asthma [34]. In Scotland with a sample of almost 1.8 million people of all ages, multimorbidity affects one in four people [10]. Multimorbidity is often met in chronic diseases and in the elderly population [12]. Studies in patients with asthma that do not exclude patients because of comorbidities are thus warranted. Studies such as the Seinäjoki Adult Asthma Study (SAAS) that evaluate the long-term prognosis of new-onset asthma diagnosed at adult age and excludes only childhood-onset patients may shed light on this matter [219].

Many of the comorbidities associated with asthma occur as cluster of diseases rather than as a single combination of asthma with a specific comorbidity. As many of the suggested mediators are common to these diseases, we propose that there may not be a single mechanism for interaction between asthma and certain comorbidity and another separate mechanism for the interaction between asthma and second comorbidity, and so forth. Instead, we think that there exists a group of mechanisms that link with each other and most link with obesity and/or components of metabolic syndrome.  Table 2 presents an overview of proposed mechanisms between asthma and certain comorbidities. Several mediators (e.g., IL-6 and ADMA) are associated with asthma and several comorbidities ranging from obesity to cardiovascular and psychiatric ones, suggesting that in addition to disease-specific mediators there may be mediators that drive the systemic inflammation playing a major role in the cluster of diseases around asthma.

These comorbidities may have common mechanisms and mediators. There exist some data that obesity and mental diseases may complicate asthma outcome, whereas data is lacking on the effects of metabolic syndrome, diabetes, and cardiovascular diseases on the outcomes of clinical asthma.

## Figures and Tables

**(a):** 

Study	Patient with asthma *n*	Setting	Country	Asthma criteria	Severity levels included	Smokers included	Other exclusion criteria	Onset of asthma (child/mixed/ adult)	Gender (Females%)	Age (mean; years)
Lessard et al., 2008 [46]	88	Cross-sectional	Canada	Confirmed diagnosis based on bronchodilator response or airway responsiveness measurements	All	No	Current smoking or ex-smoker for ≤6 mo	Mixed	68–100	32–44

Pakhale et al., 2010 [44]	346	Cross-sectional	Canada	Sequential lung testing	All	Yes (?)	Asthma ruled out using sequential testing	Mixed	66–73	41–47

Holguin et al., 2011 (SARP early-onset) [47]	543	Cross-sectional	US	A 12% increase in FEV_1_ after a short-actingbronchodilator or a 20% decrease in FEV_1_after inhalation of methacholine (PC_20_, 25 mg/mL)	All	No	Current smoking or smoking history of 5 years or more	Childhood-onset (<12 years)	49–65	26–35
Holguin et al., 2011 (SARP late-onset) [47]	506	Cross-sectional	US		All	No		Late-onset (12 years or more)	65–74	39–45

Gibeon et al., 2013 [48]	666	Cross-sectional	UK	ATS definition of refractory asthma	Severe	Yes (?)	NR	Mixed	65	45

Bruno et al. 2014 [49]	102	Cross-sectional	Italy/France	ATS 1987	Severe	No	Potential confounding diagnosis. Any persistent environmental trigger, COPD, or other differential diagnoses	Mixed	56	57

Ramasamy et al., 2014 [50]	60	Cross-sectional	India	GINA 2009	All	No	Smoker (current or ex), ICS/OCS during previous month, medication for obesity, hypertension, diabetes mellitus/CAD, unable to perform exhaled nitric oxide maneuver	Mixed	50	32–35 (inclusion 20–50 years)

Ciprandi et al., 2014 [51]	286	Cross-sectional	Italy	Documented asthma diagnosis by a specialist based on a history of intermittent wheezing in combination with reversibility to bronchodilators and/or BHR to methacholine	All	No (?)	History of lung disease other than asthma, coronary artery disease, congestive heart failure, cor pulmonale, recent asthma exacerbation or presence of acute (in the last 4 weeks) or chronic upper and/or lower respiratory infections	NR	59	48

NR = not reported, FEV_1_ = forced expiratory volume in 1 second, ATS = American Thoracic Society, SARP = the Severe Asthma Research Network, BHR = bronchial hyperresponsiveness, ICS = inhaled corticosteroid, OCS = oral corticosteroid, and CAD = coronary artery disease.

**(b):** 

	Age	Age at onset	Asthma duration	Asthma control	Exacerbations last year	Oral steroids for asthma in the previous year	Hospital admissions last year	Urgent visits to health care due to respiratory symptoms or ED visit preceding year	Hospitalization for asthma ever	Intensive care unit for asthma ever or previous year		Other
Lessard et al., 2008 [46]	*↔*	ND	*↔*	↓	ND	ND	ND	ND	ND	ND		TLC, ERV, FRC, and RV lower in obese asthmatics
Pakhale et al., 2010 [44]	↑	↑	*↔*	ND	ND	ND	*↔*	*↔*	ND	ND		Obese individuals who make urgent visits for respiratory symptoms are more likely to receive a misdiagnosis of asthma
Holguin et al., 2011, early-onset [47]	↑	*↔*	↑	ND	ND	↑	↑	↑	ND	↑		Asthmatic subjects are differentially affected by obesity based on whether they had asthma early (<12 years of age) or later in life
Holguin et al., 2011, late-onset [47]	↑	↑	*↔*	ND	ND	*↔*	(↑)	↑	ND	↑		
Gibeon et al., 2013 [48]	*↔*	*↔*	*↔*	ND	ND	↑	*↔*	*↔*	ND	*↔*		No difference in FENO or sputum eosinophils
Bruno et al., 2014 [49]	*↔*	ND	*↔*	↓	(↑)	↑	ND	ND	*↔*	*↔*		BMI represents per se a factor for the deterioration in disease control in severe asthma
Ramasamy et al., 2014 [50]	(↑)	ND	*↔*	ND	ND	ND	ND	ND	ND	ND		No difference in FENO and hsCRP
Ciprandi et al., 2014 [51]	↑	ND	ND	*↔*	ND	ND	ND	ND	ND	ND		No difference in FENO

	FEV_1_ (% pred.)	FVC (% pred.)	FEV_1_/FVC	LABA use	Blood eosinophils	Total IgE	Allergic sensitization	GERD	Diabetes mellitus	Hypertension	Obstructive sleep apnea syndrome	Anxiety/depression

Lessard et al., 2008 [46]	*↔*	*↔*	*↔*	ND	*↔*	*↔*	*↔*	ND	ND	ND	ND	ND
Pakhale et al., 2010 [44]	↓	ND	ND	ND	ND	ND	ND	↑	↑	↑	ND	ND
Holguin et al., 2011, early-onset [47]	↓	↓	↓	↑	*↔*	*↔*	*↔*	ND	ND	ND	ND	ND
Holguin et al., 2011, late-onset [47]	↓	↓	*↔*	↑	*↔*	*↔*	(↓)	ND	ND	ND	ND	ND
Gibeon et al., 2013 [48]	*↔*	↓	*↔*	ND	*↔*	↓	(↓)	↑	ND	ND	ND	ND
Bruno et al., 2014 [49]	(↓)	(↓)	*↔*	↑	*↔*	*↔*	*↔*	*↔*	↑	*↔*	↑	*↔*
Ramasamy et al., 2014 [50]	*↔*	*↔*	*↔*	ND	ND	ND	*↔*	ND	ND	ND	ND	ND
Ciprandi et al., 2014 [51]	↓	↓	↓	ND	ND	ND	↓	ND	ND	ND	ND	ND

ND = not defined; FEV_1_ = forced expiratory volume in 1 second. FVC = forced vital capacity. LABA = long-acting 2-agonist. GERD = gastroesophageal reflux disease, TLC = total lung capacity, ERV = expiratory reserve volume, FRC = functional residual capacity, RV = residual volume, FENO = forced exhaled nitric oxide, hsCRP = high sensitivity C-reactive protein, and ED = emergency department.

**Table 2:** Comorbidities and their mechanistic links to asthma.

Comorbidities and their characteristic features/mechanisms	Mediators	Link of mechanism to asthma	Ref.
*Obesity, metabolic syndrome, diabetes*			
Adverse* in utero* conditions (such as maternal obesity)		Adverse conditions may induce, for example, epigenetic changes in foetus and lead to modified activity of genes important for immune system/metabolism.	[129–133]
Systemic inflammation and inflammasome activation	IL-6, CRP, TNF-*α* IL-1*β*	IL-6 and CRP associated with reduced lung function. IL-6 may promote Th17 differentiation and severe neutrophilic asthma. IL-1*β* and IL-17 linked with development of AHR.	[138, 140, 143, 144, 154, 157]
Adipokine leptin elevated	Leptin	Airway reactivity correlated with visceral fat leptin expression. Adipokines may have direct effects on airways.	[158, 161–163]
Hyperglycemia/hyperinsulinemia	Glucose, insulin	Glucose and insulin may have direct effects on lungs promoting airway remodelling and AHR.	[179, 180, 182, 185]
Mechanical effects of obesity		Altered lung mechanics and dysfunction of peripheral airways in obese subjects: genetic properties of the airway wall may determine whether asthma develops.	[191–193]
Mitochondrial dysfunction/oxidative stress	Oxidants	Oxidative stress, for example, damages airway epithelia, modifies expression of inflammatory genes and has many deleterious effects in asthma.	[194, 199–201, 220]
Increased ADMA (inhibition of NOS)	ADMA	Decreased ratio of L-arg/ADMA was associated with worse outcome of asthma in obese patients. Effects of ADMA on asthma may be mediated by oxidants.	[202–204, 207, 209, 210]

*Coronary heart disease*			
Upregulated leukotrienes and 5-LO	LTs 5-LO	CysLTs are derived from 5-LO activity and well-known mediators of bronchoconstriction and mucus production.	[215–217]
Increased ADMA (inhibition of NOS)	ADMA	See the above effects of increased ADMA on asthma.	[207–210]

*Depression*			
Systemic inflammation and inflammasome activation	IL-6, CRP, TNF-*α*, IL-1*β*	See the above effects of systemic inflammation on asthma. Inflammatory cytokines may also generate symptoms of depression. In mice studies IL-1*β* upregulated serotonin transporter gene.	[149–151, 155, 156]
Increased ADMA (inhibition of NOS)	ADMA	See the above effects of increased ADMA on asthma.	[205–207, 209, 210]

AHR = airway hyperresponsiveness, ADMA = asymmetric dimethyl arginine, NOS = nitric oxide synthase, HPA = hypothalamic-pituitary-adrenal, 5-LO = 5-lipoxygenase, GR = glucocorticoid, L-arg = L-arginine, LT = leukotriene, cysLT = cysteinyl leukotriene, IL = interleukin, TNF = tumor necrosis factor, and CRP = C-reactive protein.
